# Freeman-Sheldon Syndrome

**Published:** 2013-01-01

**Authors:** Sajad Ahmad Salati, Mahboob Hussain

**Affiliations:** Department of Surgery, Qassim College of Medicine, Qassim University, Saudi Arabia.; Department of Radiology, Qassim College of Medicine, Qassim University, Saudi Arabia.

**Dear Sir,**

 Freeman-Sheldon syndrome (FSS) is a rare inherited form of distal arthrogryposis characterized by craniofacial deformities, camptodactyly with ulnar deviation of the fingers, and talipes equinovarus. Less than hundred cases have been reported till 2010 [1-3]. Multiple surgical interventions are needed to provide an acceptable quality of life. Anesthetic complications occur commonly [4, 5]. We present one such case that succumbed to anesthesia related complication.

A 15-month-old male child presented with feeding difficulty. The child was the only offspring, conceived and born spontaneously, after 14 years of non consanguineous marriage. There was no significant antenatal or family history of congenital anomalies. The patient had multiple admissions for evaluation of chest infection, congenital defects, and feeding difficulties. On examination, the patient had 9 kg weight. He had abnormal whistling facies including an extremely small puckered mouth; thin, pursed lips with long and well developed philtrum of upper lip, unusually prominent cheeks, and broad forehead (Fig. 1A). The child could tolerate liquid diet but there was difficulty in introducing solid foods. There were widely spaced deep set eyes with down slanting palpebral fissures and blepharosynechia. The nose was broad and saddle shaped with prominent nasal-labial folds. The ears were low set. Both upper limbs had camptodactyly with ulnar deviation of fingers and wrists with contracture of thumbs in adduction (Fig. 1B). The other significant anomalies included scoliosis of thoracic spine, deformed rib cage, bilateral talipes equinovarus and cryptorchidism (Fig. 1C). The patient was investigated (Fig. 2, 3), and referred to pediatric tertiary care facility for staged oral commissuroplasty. The patient however succumbed to arrhythmias and cardiac arrest during operation.

**Figure:**
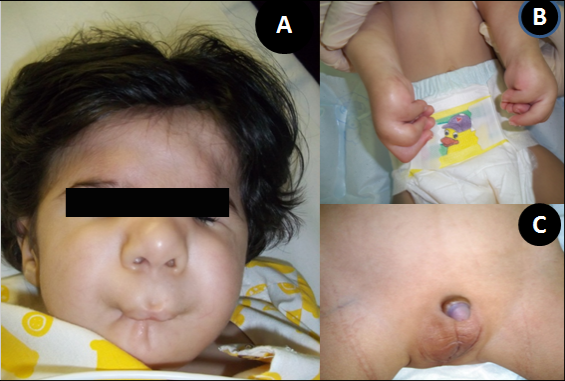
Figure 1: (A) Characteristic whistling face deformity (B) Bilateral camptodactyly (C) Cryptorchidism.

**Figure:**
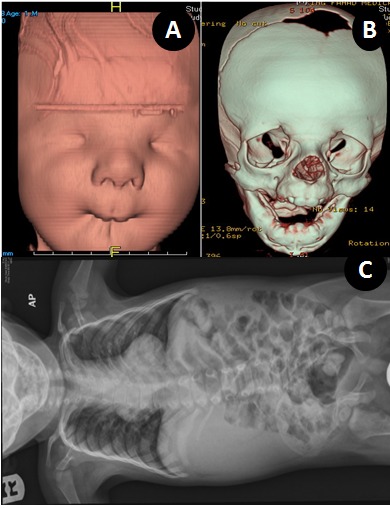
Figure 2: (A-B) X rays both hands and wrist AP views showing arthrogryposis, camptodactyly, and ulnar deviation of fingers. (C) Equinovarus deformity.

**Figure:**
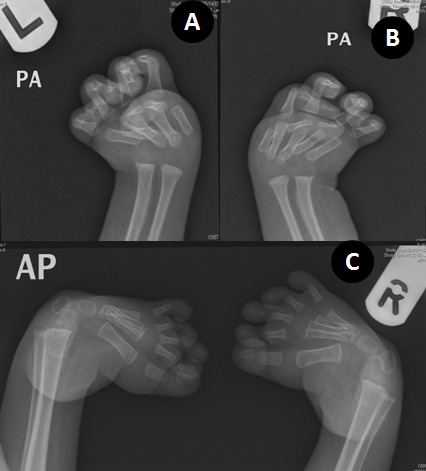
Figure 3: (A) 4D reconstructed colored CT image of face showing round forehead, prominent cheeks, broad nasal bridge, long philtrum, small nose, hypoplastic nostrils, and small mouth with whistling appearance. (B) 4D reconstructed bony image of the skull / face showing prominent forehead, patent anterior fontanel, non fused coronal suture, fused sagittal suture, hypotelorism with large orbits, long philtrum, and abnormal dentition. (C) Plain X ray chest and abdomen AP view showing hypoplastic cervical vertebral bodies, scoliosis of dorsal spine, wide ribs with narrow vertebral ends, normal lung inflation and normal bowel gas shadows.

Freeman-Sheldon syndrome (FSS) is a rare form of multiple congenital contractures (arthrogryposis) described by Freeman and Sheldon in 1938 in two unrelated subjects of opposite gender [1]. It is also called synonymously as whistling face syndrome, cranio-carpo-tarsal dysplasia syndrome, cranio-carpo-tarsal dystrophy, and distal arthrogryposis Type 2A (DA2A) [2]. 

Most cases are reported from Europe with equal gender predisposition. The syndrome is generally described as autosomal dominant [6] though a few cases of autosomal recessive transmission are also reported. More than 70% of cases occur sporadically and have no significant family history like in our case. Recent studies focus on mutations in MYH3 gene as a cause for this syndrome [6]. Biopsies of the affected facial muscles (orbicularis, masseter, buccinators and risorius) have been reported to show atrophy of the muscular fibers with abundant infiltration of adipose tissue, fibrosis, central migration of the nucleus and variation in diameter of the muscular fibers [7].

Diagnosis is made by clinical examination. Typical facial features, camptodactyly with ulnar deviation of fingers, and bilateral clubfeet are the fundamental findings in this syndrome. The other variable features could be scoliosis, articular stiffness, supra-orbital swelling, hypotrophic (myopathic) musculature, delayed milestones and short stature, hypertelorism, epicanthus, telecanthus, prominence of the supra-ciliary arches, hypoplasia of the nasal alae, low-set ears, contracture of the thumb in adduction, spina bifida occulta, and dysplasia or congenital dislocation of the hip [1-5]. 

The patients suffer from feeding difficulties, vomiting and dysphagia leading to failure to thrive and delayed development. Aspiration may lead to early mortality. The majority of cases who survive have near normal intelligence and life expectancy [2]. Dental crowding and maintenance of oral hygiene secondary to microstomia also pose a problem. The aims of treatment are to improve the skeletal deformities, respiratory function, and feeding difficulties [7]. The treatment involves multiple surgical procedures during the life time which include feeding gastrostomy, commissurotomy, dental extractions, herniotomy, spinal stabilization and management of cryptorchidism etc. The anesthetic concerns include malignant hyperthermia, arrhythmias, and difficulties with the airway intubation and intravenous access [4]. Careful pre-anesthetic evaluation is important as anesthetic complications occur in about 50% of cases as happened in the index case [5-8].

## Footnotes

**Source of Support:** Nil

**Conflict of Interest:** None declared
